# Association between the proportion of subchorionic hematoma within the gestational sac and miscarriage rate in IVF/ICSI patients

**DOI:** 10.3389/fmed.2025.1630213

**Published:** 2026-01-12

**Authors:** Panyu Chen, Yun Hu, Xiaoping Liu, Weie Zhao, Cong Fang

**Affiliations:** 1 Department of Reproductive Medicine Center, The Sixth Affiliated Hospital of Sun Yat-sen University, Guangzhou, Guangdong, China; 2 Guangdong Engineering Technology Research Center of Fertility Preservation, Guangzhou, Guangdong, China; 3 Biomedical Innovation Center, The Sixth Affiliated Hospital, Sun Yat-sen University, Guangzhou, Guangdong, China

**Keywords:** miscarriage rate, live birth rate, subchorionic hematoma, subchorionic hematoma’s proportion in gestational sac, assisted reproduction technology

## Abstract

**Background:**

The main objective of this study was to summarize and analyze the pregnancy outcomes in the assisted reproductive technology (ART) population with subchorionic hematoma (SCH) in the first trimester. Moreover, we aimed to explore the risk factors for adverse pregnancy outcomes in patients with SCH.

**Methods:**

A retrospective cohort study was conducted on *in vitro* fertilization (IVF)/intracytoplasmic sperm injection (ICSI) patients who achieved pregnancy between February 2017 and November 2022 at the Reproductive Medicine Center of the Sixth Affiliated Hospital of Sun Yat-sen University. Eligible patients were divided into two groups according to whether SCH was detected.

**Results:**

After appropriate screening, 275 patients with SCH and 336 patients without SCH were enrolled. Patients with SCH had a significantly higher miscarriage rate than patients without SCH (13.5% vs. 8.3%, *p* = 0.04). Miscarriage patients in the SCH group had more embryos transferred (*p* = 0.04), earlier detection of SCH (*p* = 0.002), and a smaller gestational sac (GS)’s area (*p* = 0.03). Moreover, the proportion of SCH in the GS was a statistically significant risk factor for miscarriage (*p* = 0.016). The miscarriage rate progressively increased with increasing SCH’s proportion in the GS (*p* for trend: 0.003).

**Conclusion:**

SCH increased miscarriage rate in the ART population. Patients with SCH combined with miscarriages had more embryos transferred, earlier detection of SCH, and smaller GS areas when SCH was first detected. Multivariate logistic regression analysis showed that the SCH proportion in the GS was a statistically significant risk factor for SCH associated with miscarriage. The miscarriage rate increased progressively with the SCH proportion in the GS.

## 1 Introduction

Subchorionic hematoma (SCH) is a frequently observed finding during routine ultrasound examinations in early pregnancy. Epidemiological studies have reported an incidence ranging from 1.7 to 18.2% (1, 2). Since its first description in 1981, the clinical significance of SCH has remained unclear (3). Previous studies have yielded conflicting results; for instance, Al-Memar et al. and Mackenzie et al. reported that SCH detected before 14 weeks of gestation was not associated with pregnancy loss before 20 weeks (4) or with adverse pregnancy outcomes after 20 weeks (5). In contrast, Xiang et al. found that SCH significantly increased the risk of late-pregnancy complications such as preterm birth, preeclampsia, and postpartum hemorrhage (1), while Tom Farrell observed an association between SCH and higher miscarriage rates (6).

The majority of existing studies have focused primarily on the relationship between SCH and various pregnancy outcomes; however, few studies have addressed the potential heterogeneity among SCH cases—such as whether hematomas of different sizes affect outcomes differently. Moreover, the spatial relationship between SCH and the gestational sac (GS), and its possible relevance to pregnancy outcomes, has not been thoroughly investigated. Another limitation is the lack of research identifying specific risk factors for adverse outcomes in pregnancies complicated by SCH.

Our study aimed to address these gaps by analyzing pregnancy outcomes in an assisted reproductive technology (ART) population with SCH. We aimed to evaluate how various SCH characteristics influence adverse outcomes, identify risk factors within this population, and explore previously overlooked associations—particularly those involving SCH size and its spatial relationship to the GS.

## 2 Object and method

### 2.1 Study design

This retrospective cohort study was approved by the Reproductive Ethics Committee of the Sixth Affiliated Hospital of Sun Yat-sen University. All research was performed in accordance with relevant guidelines and regulations. Meanwhile, obtaining informed consent from each patient was not feasible due to the study’s retrospective nature. According to the Declaration of Helsinki, if obtaining consent is impossible or impractical, research can proceed only after review and approval by a research ethics committee. In this study, informed consent was waived by the Ethics Committee of the Sixth Affiliated Hospital of Sun Yat-sen University (2017ZSLYEC-016S) because it involves a retrospective review of existing data from standard patient care, and no patient information was disclosed. In this retrospective cohort study, patients who underwent IVF/ICSI at the Reproductive Medicine Research Center of the Sixth Affiliated Hospital of Sun Yat-sen University from February 2017 to November 2022 and achieved a singleton pregnancy were retrospectively analyzed, and transvaginal ultrasound results between 5 and 11 weeks of gestation were collected from these patients. They were divided into two groups according to the presence or absence of SCH, and their pregnancy outcomes were compared. Moreover, risk factors for patients with SCH who experienced miscarriage were explored.

### 2.2 Patient cohort

All patients who underwent IVF/ICSI from February 2017 to November 2022, achieved a singleton pregnancy, and underwent transvaginal ultrasound at our center during the first trimester were included. Among these patients, the following patients were excluded before the analysis: (1) those who underwent induction of labor in the second trimester due to fetal abnormalities, abnormal fetal appendages, or preterm premature rupture of membranes before 28 weeks of gestation; (2) patients with anticoagulant or aspirin use during early pregnancy; (3) patients with uterine anomalies, including uterine fibroids and adenomyosis detected by ultrasound, and genital tract abnormalities, including uterine, cervical, or vaginal abnormalities; (4) patients with genital tract infections during the first trimester; (5) patients with rheumatic immune diseases or who had recurrent miscarriages; (6) patients who had spontaneous abortion due to cervical incompetence; and (7) patients who had undergone cesarean delivery and had a uterine scar diverticulum. We divided patients into two groups according to the presence or absence of SCH, and patients with no difference in baseline conditions were further screened for comparison.

### 2.3 Ovarian stimulation, oocyte insemination, and embryo transfer

Patients were stimulated, monitored, and managed according to our center’s clinical protocols, as previously described (7). Conventional IVF or ICSI was performed according to the sperm parameters. Embryo culture, selection, cryopreservation/thawing, and transfer were carried out according to previously described protocols (7). To minimize the effect of embryo quality on the pregnancy outcomes in this study, we graded the quality of transferred embryos as follows: single or two good-quality embryos transferred were defined as grade A; one good-quality embryo and one poor-quality embryo transferred were defined as grade B; and all transferred embryos of poor quality were defined as grade C. The criteria for good-quality embryos are referenced in the literature (7). Luteal support was given uniformly from the day of egg retrieval in fresh cycles: Duphaston (dydrogesterone, Abbott Laboratories) 10 mg orally twice/day and Utrogestan (Besins) 200 mg vaginally twice/day. For the frozen embryo transfer cycle, luteal support was given uniformly from the day of ovulation in natural cycles: Duphaston 10 mg orally twice/day. For hormone replacement cycles, Progynova (Bayer) 3 mg twice/day, was started on the day of endometrial transformation, with Duphaston 10 mg orally twice/day and Utrogestan 200 mg vaginally twice/day added. All medications were continued for 17 days. Patients continued luteal support after confirmation of biochemical pregnancy via a blood β-HCG test 14 or 12 days after embryo transfer.

### 2.4 Transvaginal ultrasound examination

All pregnant women included in the study underwent transvaginal two-dimensional (2D) ultrasound from 5 weeks to 11 weeks of gestation to assess the location of the pregnancy (intrauterine or extrauterine), the size of the GS, the presence or absence of the yolk sac, the embryo, the fetal heart, the size of the embryo, the presence of uterine abnormalities such as fibroids and adenomyosis, the presence of an SCH next to the GS, and the size of the SCH. The sonogram of SCH appeared as a collection of fluid between the GS and the uterine wall and could take many shapes, including crescentic or circular, with no blood flow signal detected by color Doppler and energy Doppler. The GS was clearly visualized in the sagittal plane; the longest and perpendicular width diameters between the inner walls of the sac were measured and multiplied to obtain the area of the GS. The SCH was visualized in the sagittal plane; the longest and perpendicular widths of the SCH were measured and multiplied to obtain its area. All measurements were in millimeters, and the area was in square millimeters.

### 2.5 Outcome measurements

The main outcome of this study was the miscarriage rate and the live birth rate. Miscarriage was defined as spontaneous abortion before 28 weeks of gestation. Live birth was defined as an individual newborn after 28 weeks of gestation. Both rates were calculated based on the total number of pregnant women included in this study, and miscarriage and live birth rates were thus derived. Preterm birth is defined as labor between 28 weeks and less than 37 weeks of gestation.

### 2.6 Data analysis and statistics

According to preliminary data from our center, the miscarriage rate during early pregnancy was approximately 5% in women under 35 years receiving IVF/ICSI treatment without SCH and approximately 13% in those with sonographically detected SCH. Based on these findings, the expected miscarriage rates for the non-SCH and SCH groups were set at 5 and 13%, respectively. With a two-sided significance level of 0.05, statistical power of 80%, and 1:1 group allocation, sample size calculation was performed using PASS software. The results indicated that a minimum of 197 participants per group (total ≥394) were required. Our final matched cohort of 705 patients (SCH: 358; non-SCH: 347) therefore satisfied this requirement.

All statistical analyses of data were performed using SPSS 26.0. To minimize potential confounding factors and ensure comparability between the SCH and non-SCH groups, we performed propensity score matching (PSM) at a 1:2 ratio (nearest-neighbor matching with a caliper width of 0.02). The propensity score was estimated using a logistic regression model that included the following covariates, selected for their known or potential influence on pregnancy outcomes: age, infertility type, infertility duration, number of previous abortions, etiology, Anti-Müllerian Hormone level, basal Follicle-Stimulating Hormone level, type of cycle (fresh/frozen), type of embryos transferred (D3/D5), number of embryos transferred, and grade of embryos transferred. The balance of covariates before and after matching was assessed using standardized mean differences (SMD), with an SMD < 0.1 indicating good balance. Before PSM, there were some differences in baseline characteristics between the initial SCH and non-SCH cohorts (see Supplementary Table 1). After PSM, all 611 patients (275 with SCH, 336 without SCH) in the matched cohorts were well-balanced across all included covariates (Table 1). Pearson’s chi-squared test or Fisher’s exact test was used for qualitative variables, and Student’s *t*-test or the Mann–Whitney test was used for quantitative variables, as appropriate. Baseline characteristics are presented as means ± standard deviations or medians with interquartile ranges, while categorical data are presented as counts and percentages. Two-sided *p*-values of <0.05 were considered statistically significant. In the analysis of risk factors for SCH combined with miscarriage, we included the variables whose *p* < 0.1, that is, infertility factor, number of embryos transferred, week of gestation when SCH was first detected, area of the GS and SCH’s proportion in the GS, in a multivariate logistic regression model, and adjusted ORs and 95% CIs were obtained using the enter method. To explore the association between the SCH’s proportion in the GS and the miscarriage rate/live birth rate, patients with single GS and SCH were extracted for further analysis. Rates among different groups were compared using the chi-squared test. The *p*-value for trend was calculated using the linear-by-linear method.

**Table 1:** Baseline characteristics of patients.

Characteristics	SCH	No SCH	*p*
*n*	275	336	
Age	32.1 ± 4.1	32.3 ± 4.1	0.44
Infertility type			0.59
Primary infertility	133 (48.4)	168 (50.0)	
Secondary infertility	142 (51.6)	168 (50.0)	
Infertility duration	4.1 ± 2.8	4.2 ± 2.9	0.51
Number of abortions	0.5 ± 0.9	0.6 ± 0.9	0.52
Etiology			0.74
Unexplained infertility	7 (2.6)	9 (2.7)	
Polycystic Ovary Syndrome	9 (3.3)	12 (3.6)	
Recurrent Intrauterine Insemination failure	5 (1.8)	6 (1.8)	
Low ovarian reserve	8 (2.9)	20 (6.0)	
Male factor	39 (14.2)	33 (9.8)	
Ovulatory dysfunction	3 (1.1)	3 (0.9)	
Tubal factor	74 (26.9)	97 (28.9)	
Sexual intercourse disorders	0	1 (0.3)	
Endometriosis	5 (1.8)	5 (1.5)	
Genetic factor	1 (0.4)	2 (0.6)	
Multiple factors	124 (45.1)	148 (44.1)	
AMH	3.6 ± 3.4	4.0 ± 3.5	0.55
Basal FSH	6.0 ± 2.8	6.2 ± 2.8	0.69
Type of cycle			0.12
Fresh cycle	67 (24.4)	68 (20.2)	
Frozen cycle	206 (74.9)	268 (79.8)	
Mixed cycle	2 (0.7)	0	
Type of embryos transferred			0.22
D3	105 (38.2)	103 (30.7)	
D5	170 (61.8)	233 (69.4)	
Number of embryos transferred	1.6 ± 0.5	1.6 ± 0.5	0.31
Grade of embryos transferred			0.13
A	233 (84.7)	286 (85.1)	
B	32 (11.6)	28 (8.3)	
C	10 (3.7)	22 (6.6)	

“Mixed cycle” refers to a treatment cycle in which, following egg retrieval, both thawed frozen embryos and fresh embryos are transferred simultaneously.

## 3 Results

**Figure 1:**
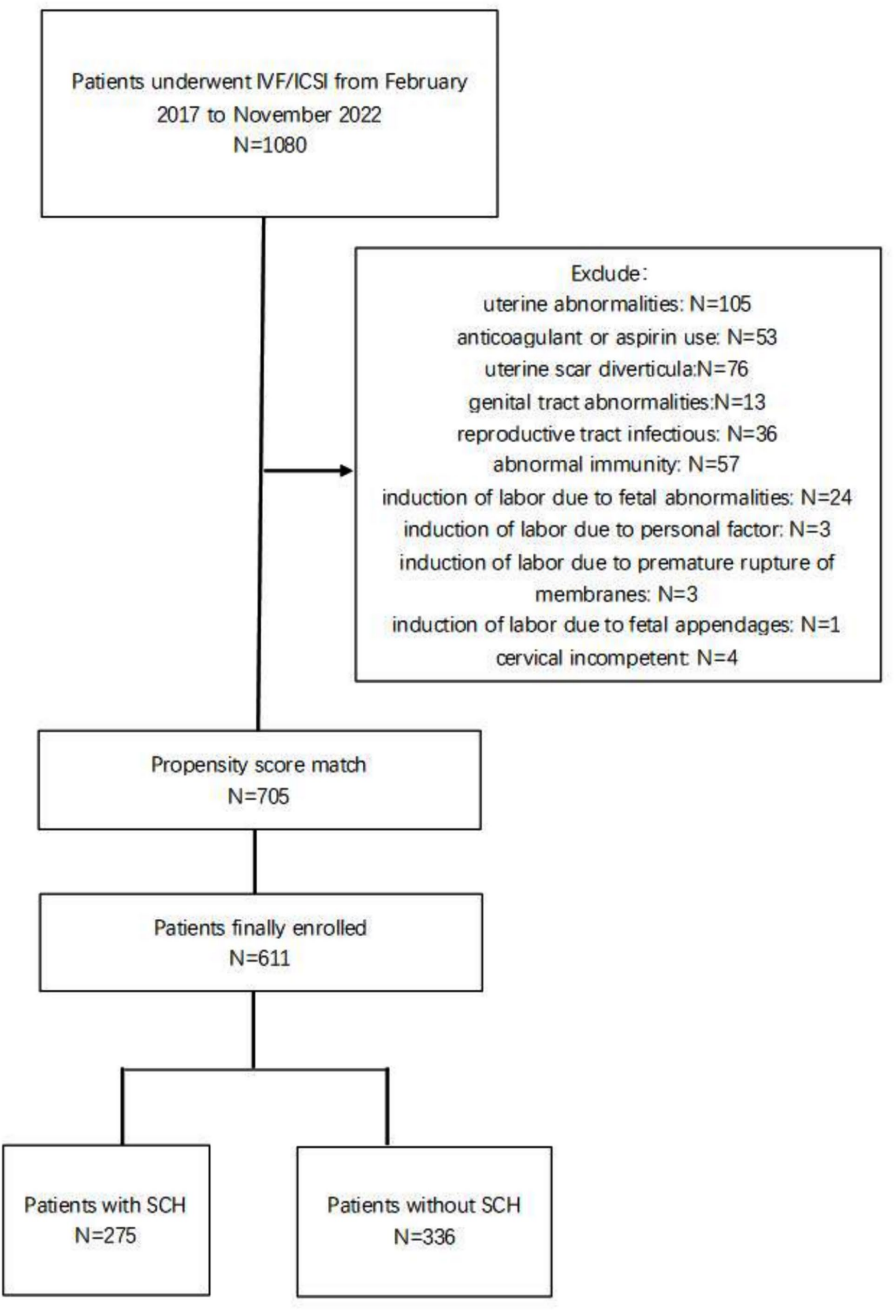
Flowchart of the study design.

The process of cohort selection is detailed in Figure 1. We collected a total of 1,080 pregnancy patients who underwent IVF/ICSI and had their first transvaginal ultrasound examination at our center during the first trimester, from February 2017 to November 2022. A total of 375 patients were excluded from the study for the following reasons: 105 patients with uterine abnormalities such as uterine fibroids and adenomyosis identified by ultrasound; 53 patients with anticoagulant or aspirin use during early pregnancy; 76 patients who had cesarean delivery with confirmed uterine scar diverticula; 13 patients with genital tract abnormalities; 36 patients with reproductive tract infections in the first trimester, 57 patients with abnormal immune indices, rheumatic immune diseases, or recurrent miscarriages; 24 patients who underwent induction of labor in the second trimester due to fetal abnormalities; 3 patients who underwent induction of labor in the second trimester due to personal factors; 4 patients with spontaneous abortions due to cervical incompetent; 3 patients who underwent induction of labor in the second trimester due to premature rupture of membranes; and 1 patient who underwent induction of labor in the second trimester due to fetal appendages. A total of 705 patients finally met the inclusion criteria. Among the 705 patients who met the inclusion criteria, 358 were diagnosed with SCH, yielding an incidence rate of 50.8% (358/705) in our study population.

We used propensity scores to match and select a total of 611 patients from the two groups who had similar baseline conditions. Of these, 275 patients were found to have SCH during transvaginal ultrasound, and 336 patients did not have SCH. Comparison of basic conditions is shown in Table 1. There were no significant differences with respect to their age, type of infertility, years of infertility, number of previous miscarriages, infertility factors, AMH, basal FSH, cycle type, type, number, and grade of embryos transferred. Comparison of pregnancy outcomes is presented in Table 2. The miscarriage rate was higher in patients with SCH than in those without SCH, with a statistically significant difference (13.5% vs. 8.3%, *p* < 0.05); the live birth rate was significantly higher in the non-SCH group than in the SCH group (91.7% vs. 86.5%, *p* = 0.04). There was no significant difference between preterm birth rates, neonatal gestational weeks, and birth weight between the two groups.

**Table 2:** Comparison of pregnancy outcomes.

Pregnancy outcome	SCH	No SCH	*p*
*n*	275	336	
Miscarriage	37 (13.5)	28 (8.3)	0.04
Preterm birth	3 (1.1)	5 (1.5)	0.74
Live birth	238 (86.5)	308 (91.7)	0.04
Gestational week	38.5 ± 0.6	38.6 ± 0.4	0.32
Birth weight (g)	3210.8 ± 243.6	3,197 ± 215.4	0.25

Next, we compared the baseline characteristics of patients with SCH who experienced miscarriages to those who did not. Statistically significant differences were observed between the miscarriage and the non-miscarriage groups in the number of embryos transferred and the gestational week at which SCH was first detected. The results are shown in Table 3. Multivariable analysis showed that after adjustment for potential confounders, the proportion of SCH in the GS was a statistically significant risk factor for miscarriage in patients undergoing IVF/ICSI (*p* = 0.016, OR = 1.003, 95%CI:1.001–1.005), as shown in Table 4.

**Table 3:** Comparison of baseline characteristics between SCH patients with miscarriage and without miscarriage.

Baseline characteristics	Miscarriage	No miscarriage	*P*
*n*	37	238	
Age	32.4 ± 4.2	32.1 ± 4.1	0.59
Infertility type			0.58
Primary infertility	21 (56.8)	122 (51.3)	
Secondary infertility	16 (43.2)	116 (48.7)	
Years of infertility	4.0 ± 3.1	4.2 ± 2.8	0.73
Number of abortion	0.7 ± 1.1	0.5 ± 0.8	0.40
Etiology			0.12
Unexplained infertility	3 (8.1)	5 (2.0)	
PCOS	2 (5.4)	7 (2.9)	
Recurrent IUI failure	0	5 (2.1)	
Low ovarian reserve	0	8 (3.4)	
Male factor	5 (13.5)	33 (13.9)	
Ovulatory dysfunction	1 (2.7)	2 (0.8)	
Tubal factor	6 (16.2)	68 (28.6)	
Endometriosis	2 (5.4)	3 (1.3)	
Genetic factor	0	1 (0.4)	
Multiple factors	18 (48.7)	106 (44.5)	
AMH	3.9 ± 3.2	4.0 ± 3.5	0.90
Basal FSH	5.5 ± 3.1	6.1 ± 2.7	0.29
Type of cycle			0.47
Fresh cycle	6 (16.2)	61 (25.6)	
Frozen cycle	31 (83.8)	175 (73.5)	
Mixed cycle	0	2 (0.8)	
Type of embryos transferred			0.32
D3	13 (35.1)	92 (38.7)	
D5	24 (64.9)	146 (61.3)	
Number of embryos transferred	1.7 ± 0.5	1.5 ± 0.5	0.04
Grade of embryos transferred			0.48
A	34 (91.9)	199 (83.6)	
B	3 (8.1)	29 (12.2)	
C	0	10 (4.2)	
Gestation week when SCH was first detected	6.2 ± 1.0	6.8 ± 1.2	0.002
Area of the GS (mm^2^)	505.1 ± 423.4	710.9 ± 526.0	0.03
Area of SCH (mm^2^)	414.9 ± 526.5	295.1 ± 341.2	0.19
SCH’s proportion in the GS	155.1 ± 279.0%	63.0 ± 101.8%	0.05

**Table 4:** Logistic model for risk factors for miscarriage in SCH.

Risk factor	b	S.E.	Wald *χ*^2^	*p*	OR	95%CI
Constant	−0.620	0.925	0.449	0.503	0.538	
Etiology			7.754	0.458		
Number of embryos transferred	0.686	0.422	2.635	0.105	1.985	(0.867–4.542)
Gestation week			5.951	0.429		
Area of the GS (mm^2^)	0.000	0.001	0.329	0.566	1.000	(0.999–1.002)
SCH’s proportion in the GS	0.003	0.001	5.773	0.016	1.003	(1.001–1.005)

**Figure 2:**
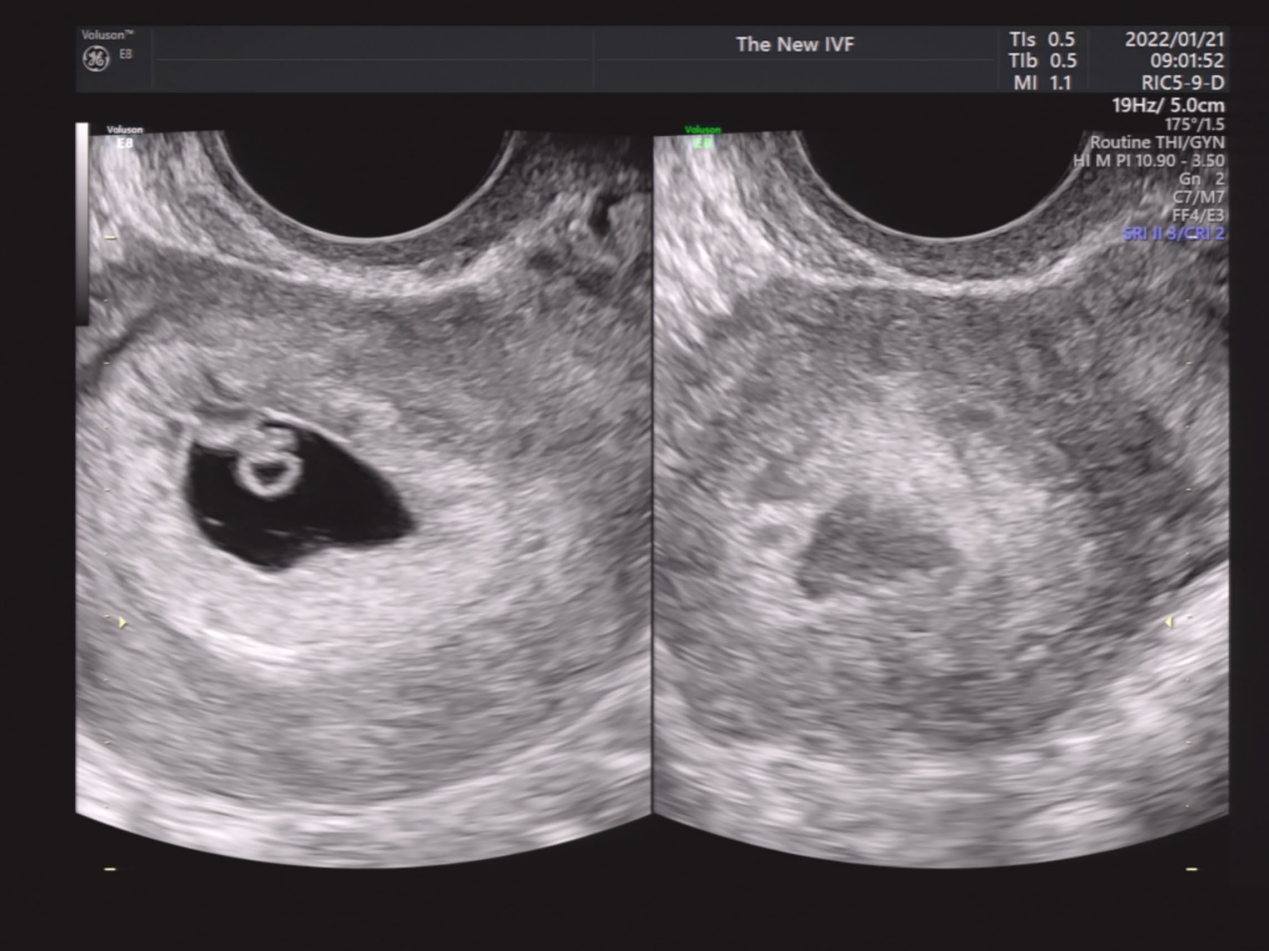
A patient with an SCH whose proportion was less than 50% of the GS.

**Figure 3:**
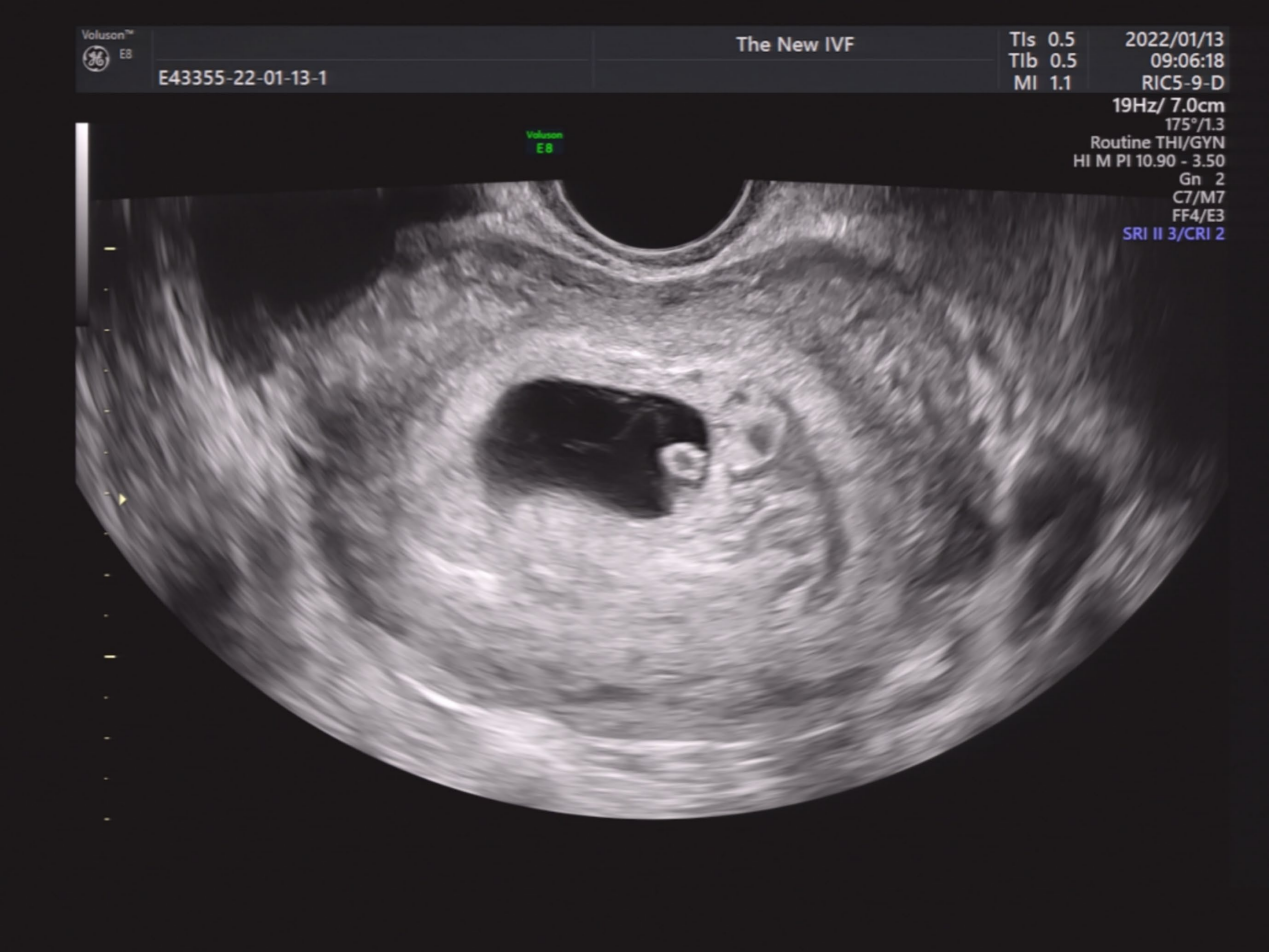
A patient with an SCH whose proportion was between 50 and 100% of the GS.

**Figure 4:**
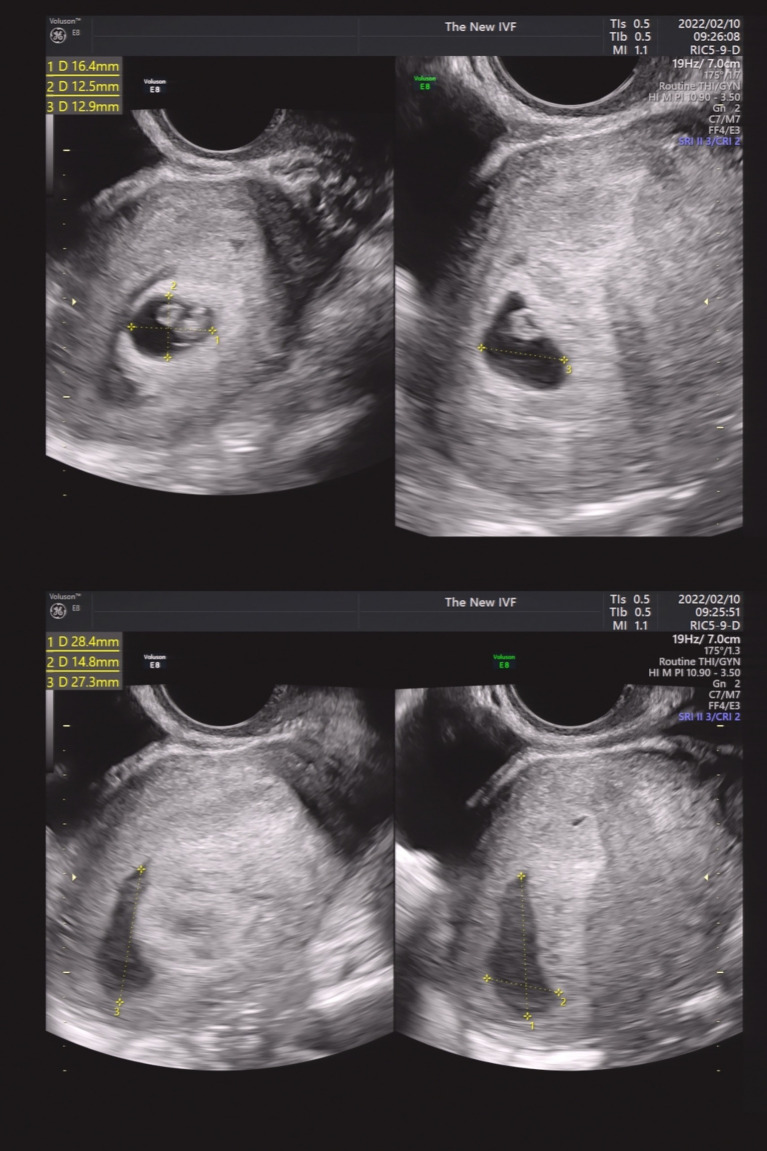
A patient with an SCH whose proportion was between 100 and 500% of the GS.

**Figure 5:**
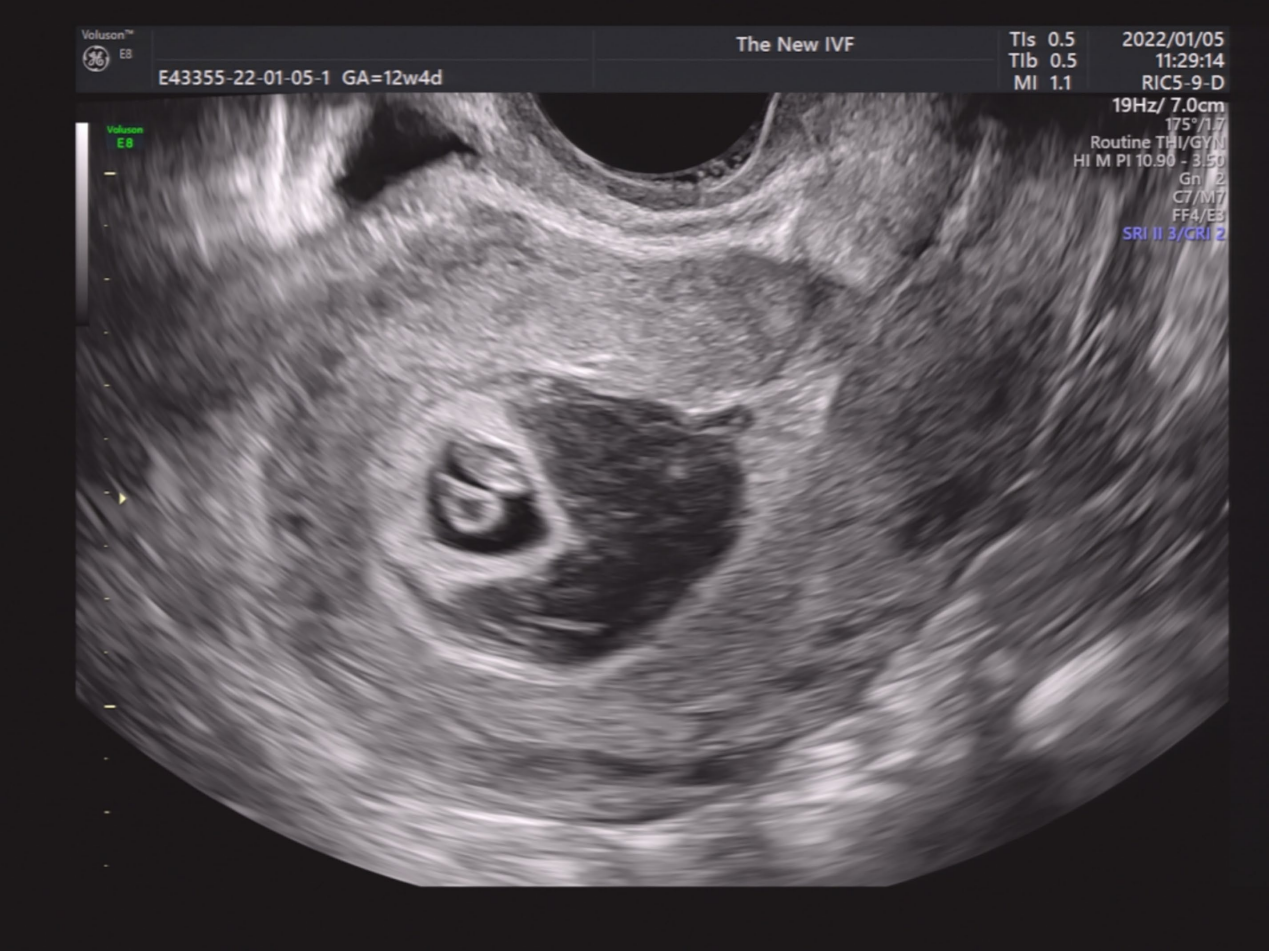
A patient with an SCH whose proportion was larger than 500% of the GS.

We then performed a subgroup analysis of SCH patients with single GS. Patients were divided into four groups according to the SCH’s proportion in the GS. As shown in Figures 2–5, Figure 2 shows a patient with an SCH whose proportion was less than 50% of the GS; Figure 3 shows that SCH’s proportion was between 50 and 100% of the GS; Figure 4 show that SCH’s proportion was between 100 and 500% of the GS; and Figure 5 shows that SCH’s proportion was larger than 500% of the GS. A significant association was observed between SCH’s proportion in the GS and the miscarriage rate. A larger proportion was associated with a higher miscarriage rate (*p* for trend: 0.003). The results are shown in Table 5.

**Table 5:** General patients’ characteristics according to the proportion of SCH.

Characteristics	SCH’s proportion in the GS					
	<50%	50%≤, <100%	100%≤, <500%	≥500%	*p*	*p* for trend
SCH’s proportion in the GS, Median (range)	16.8% (1.8–49.7%)	65.7% (53.0–95.0%)	175.3% (100.0–437.5%)	1,000% (594.2–1155.6%)		
*n*	181	44	45	5		
Age	32.4 ± 4.2	32.4 ± 4.4	30.8 ± 3,3	31 ± 3.7	0.131	
Infertility duration	4.2 ± 2.9	3.6 ± 2.4	3.8 ± 3.0	4.0 ± 2.0	0.536	
Infertility type					0.875	
Primary infertility	97 (53.6)	23 (52.3)	21 (46.7)	2 (40.0)		
Secondary infertility	84 (46.4)	21 (47.7)	24 (53.3)	3 (60.0)		
AMH	4.1 ± 3.5	3.2 ± 2.5	4.2 ± 3.9	2.6 ± 2.1	0.311	
Basal FSH	5.9 ± 2.8	5.9 ± 2.9	6.4 ± 2.5	6.5 ± 4.9	0.736	
No. of previous abortions					0.053	
0	118 (65.2)	31 (70.5)	31 (68.9)	2 (40.0)		
1	39 (21.5)	13 (29.5)	14 (31.1)	0		
2 or more	24 (13.3)	2 (4.5)	4 (8.9)	3 (60.0)		
Cycle type					0.012	
Fresh	33 (18.2)	16 (36.4)	17 (37.8)	1 (20.0)		
Frozen	147 (81.2)	18 (63.6)	27 (60.0)	4 (80.0)		
Mixed	1 (0.6)	0	1 (2.2)	0		
Type of embryos transferred					0.435	
D3	64 (35.4)	20 (45.5)	18 (40.0)	3 (60.0)		
D5	117 (64.6)	24 (54.5)	27 (60.0)	2 (40.0)		
Grade of embryos transferred					0.925	
A	153 (84.5)	39 (88.6)	36 (80.0)	5 (100.0)		
B	21 (11.6)	4 (9.1)	7 (15.6)	0		
C	7 (3.9)	1 (2.3)	2 (4.4)	0		
No. of embryos transferred	1.5 ± 0.5	1.6 ± 0.5	1.5 ± 0.5	2 ± 0	0.223	
Gestational week of the first detection of SCH					0.189	
5–6	20 (11.1)	3 (6.5)	7 (14.3)	1 (20.0)		
6–7	60 (33.2)	16 (36.4)	23 (51.1)	4 (80.0)		
7–8	48 (26.5)	11 (25.0)	11 (24.4)	0		
8–9	33 (18.2)	12 (27.3)	2 (4.4)	0		
9–10	18 (9.8)	2 (4.5)	2 (4.4)	0		
10–11	1 (0.6)	0	0	0		
11–12	1 (0.6)	0	0	0		
Area of SCH (mm^2^)	134.5 ± 107.1	443.7 ± 360.7	773.6 ± 426.6	1381.4 ± 382.1	0.000	
Abortion	20 (11.0)	6 (13.6)	7 (15.6)	4 (80.0)	0.004	0.003

## 4 Discussion

Our study revealed a significantly increased miscarriage rate in ART patients diagnosed with first-trimester SCH on ultrasound, consistent with several previous reports (6, 8–10). Tom Farrell et al. observed higher pregnancy loss among women presenting with vaginal bleeding accompanied by early-pregnancy SCH. Similarly, a 1996 study by Ball et al. (11) revealed that ultrasound-confirmed SCH is correlated with elevated risks of miscarriage, stillbirth, placental abruption, and preterm delivery. Peixoto et al. (10) also reported that SCH is associated not only with an enlarged mean yolk sac diameter but also with an increased risk of miscarriage. Further corroborating these findings, Heller et al. (3) confirmed the relationship between SCH and a higher incidence of miscarriage. More recently, a 2022 study by Elmas et al. reported that first-trimester SCH increases the risk of miscarriage by 1.58-fold in women with threatened abortion, although it does not influence delivery complications in ongoing pregnancies (12).

Our findings revealed a significant association between SCH and increased miscarriage rates, in contrast with several previous studies in ART populations. Zhou et al. (13) reported no significant difference in miscarriage rates between the SCH and non-SCH groups, despite observing lower birth weights in SCH-associated singleton pregnancies. Similarly, Yin et al. (14) conducted a large propensity score-matched cohort study and found that first-trimester SCH detected at 6–8 weeks of gestation was not associated with adverse pregnancy outcomes, including miscarriage, following fresh embryo transfers. They identified vaginal bleeding rather than hematoma presence as the primary risk factor for pregnancy loss. Consistent with these reports, Al-Memar et al. (5) concluded that early-pregnancy SCH increases preterm birth risk but not miscarriage risk, even after adjusting for vaginal bleeding, lower abdominal pain, and gestational age, while Pillai et al.’s (4) meta-analysis found SCH to be a poor predictor of miscarriage.

The discrepancy between our results and these earlier findings may be attributed to key methodological differences. Unlike previous studies that primarily evaluated SCH based on its mere presence or absolute size, our study introduced and quantified the “SCH proportion in GS” as a novel metric. This parameter may more sensitively reflect the relative mechanical and functional compromise of the gestational sac, potentially identifying a clinically significant SCH subset previously overlooked. Furthermore, our implementation of strict exclusion criteria minimized confounding from other factors affecting pregnancy outcomes, allowing for a more isolated assessment of SCH-specific risk.

Ultrasonographically detected SCH in early pregnancy is a common clinical finding, with reported incidence rates ranging from 1.3 to 13.5% (15, 16). Establishing the relationship between SCH and pregnancy outcomes is therefore of significant clinical importance. Given the multifactorial etiology of miscarriage, isolating the specific contribution of SCH requires rigorous control of potential confounding factors. In the present study, after implementing strict exclusion criteria to minimize the influence of other determinants of pregnancy outcomes, SCH remained independently associated with an increased miscarriage rate. Furthermore, accumulating evidence indicates that first-trimester SCH significantly elevates the risk of various obstetric complications, including gestational hypertension, preeclampsia, postpartum hemorrhage, preterm birth, and placenta previa (5, 9, 17). These findings underscore the importance of comprehensive early-pregnancy evaluation in women with SCH to identify and address potential contributing factors to pregnancy loss, such as genital tract infections and immunological abnormalities. Throughout the second and third trimesters, close monitoring of maternal and fetal wellbeing is warranted, along with proactive measures to prevent obstetric complications.

Furthermore, while our study noted a tendency toward larger SCH area in patients who miscarried compared to those with ongoing pregnancies, this difference did not reach statistical significance. This observation aligns with the report by Dongol et al. (15), which indicated that an SCH area exceeding 20 cm^2^ was associated with an increased likelihood of miscarriage. To better evaluate the clinical relevance of hematoma size in relation to gestational development, we introduced the concept of the “SCH proportion in GS.” This metric was significantly higher in patients who experienced miscarriage, and the difference was statistically significant. Multivariable logistic regression analysis, adjusted for factors with *p* < 0.1, confirmed that the SCH proportion in the GS remained an independent risk factor for miscarriage. This finding suggests that relying solely on the presence or absolute size of SCH in clinical practice may lead to incomplete risk assessment. Consequently, evaluation should also consider the size of the gestational sac and the relative proportion of SCH within it. This is particularly relevant for pregnant women with a small gestational sac during early pregnancy, in whom even a small SCH may exert a disproportionately high relative impact and should not be overlooked. However, the clinical implications of the SCH proportion in the GS require careful interpretation. With an odds ratio of 1.003, each unit increase in SCH proportion corresponds to only a minimal increase in absolute miscarriage risk. Therefore, although statistically significant in our cohort, this parameter alone is unlikely to serve as a strong standalone predictor of miscarriage. Its potential clinical value may instead lie as one component within a comprehensive risk assessment model. Integrating SCH proportion with other established risk factors—such as maternal age, history of threatened abortion, and prior obstetric outcomes—could enhance early risk stratification.

Although subgroup analysis indicated a trend toward higher miscarriage rates with increasing SCH proportion, these results must be interpreted cautiously. Specifically, the subgroup with an SCH proportion ≥500% included only five patients, which considerably limits the statistical reliability and generalizability of findings in this category. Therefore, the results from this subgroup should be regarded as preliminary. Further multicenter studies with larger sample sizes are needed to validate whether extremely high SCH proportions significantly increase miscarriage risk and to better quantify their effect.

In fact, it has been suggested that the area of SCH should be calculated as early as 1986 (18). Bennett et al. (2) found that an SCH area of two-thirds or more of the GS was enough to predict miscarriage. A study by Heller et al. (3) evaluated the predictive effect of different methods of SCH assessment on pregnancy outcomes during early pregnancy. They also found that the method proposed by several studies (16, 18–20), which estimates the ratio of area of SCH to the GS based on the size of GS, was the most accurate predictor of pregnancy outcomes. As the proportion of SCH in GS gradually increased, the miscarriage rate gradually increased too. However, this assessment does not accurately measure the diameter of the SCH area. They estimated the size of SCH based solely on the examiner’s visualization, which is too subjective. In our study, the length and width of SCH and GS were accurately measured, which provided relatively objective data.

The precise mechanism remains unclear; however, the presence of a hematoma is hypothesized to directly compromise placental development and function through several pathways: (1) physical separation of the chorion from the decidua, impairing nutrient and gas exchange; (2) triggering a local inflammatory response and thrombin generation, which could lead to placental ischemia and dysfunction; and (3) potentially inducing uterine irritability and contractions.

From a clinical perspective, our findings that the proportion of SCH within the GS is a quantifiable risk factor—even with a modest effect size—suggest that it should not be used in isolation. Rather, clinicians can integrate this metric into a broader risk assessment strategy. For instance, patients with a large SCH proportion (e.g., ≥50%), particularly those with additional risk factors such as vaginal bleeding or a history of pregnancy loss, may warrant closer monitoring. This could include more frequent ultrasounds to track hematoma resolution and fetal wellbeing, alongside heightened vigilance for signs of threatened abortion.

Regarding interventions, while there is no universally accepted protocol to prevent miscarriage in women with SCH, potential management strategies based on common clinical practices include: (1) activity restriction or pelvic rest to reduce mechanical irritation, (2) optimization of luteal phase support with progesterone supplementation, aiming to enhance endometrial stability, and (3) in cases with co-existing risk factors (e.g., thrombophilia), consideration of low-dose aspirin or anticoagulant therapy, although this remains highly individualized and requires further investigation. Ultimately, the prognostic value of SCH proportion should be validated in larger, prospective studies focused on developing and testing integrated management protocols.

Our study also revealed that the number of embryos transferred and the gestational week at which SCH was first detected were significantly associated with miscarriage among SCH patients. Specifically, a higher number of embryos transferred may be linked to larger SCH size or more pronounced bleeding tendencies, potentially due to altered endometrial receptivity or increased hormonal stimulation. However, Asato (2014) reported that the number of embryos transferred showed no significant correlation with the occurrence of SCH (21). Moreover, earlier detection of SCH might reflect more severe or earlier-onset placental instability, which could contribute to a higher risk of miscarriage. These findings highlight the importance of closely monitoring patients with multiple embryo transfers and those diagnosed with SCH at an earlier gestational age.

This study has several limitations. First, its single-center retrospective design may introduce inherent selection bias and restrict the generalizability of the findings. Second, the sample size, although sufficient for primary outcomes, limited the statistical power of subgroup analyses—particularly in groups with extreme SCH proportions, such as the ≥500% subgroup (*n* = 5), where results should be interpreted with caution. Third, while miscarriage and live birth rates were evaluated as primary endpoints, we did not track other obstetric complications such as preterm birth, preeclampsia, or placental abnormalities, which might provide further insights into the broader clinical implications of SCH. Fourth, potential unmeasured confounders—including body mass index, lifestyle factors, detailed semen parameters, and undetected genetic or thrombophilic disorders—were not adjusted for (22), possibly influencing the outcomes. Finally, inter-observer variability in ultrasound-based SCH measurements, although mitigated by standardized protocols, may still affect classification consistency. Future multicenter prospective studies with larger cohorts and more comprehensive outcome collection are warranted to validate and extend our findings.

In conclusion, our study demonstrated that SCH is associated with an increased risk of miscarriage in patients undergoing ART. Those who experienced miscarriage in the presence of SCH were characterized by a higher number of embryos transferred, earlier detection of SCH, and a smaller GS at the time of SCH diagnosis. Multivariate logistic regression identified the proportion of SCH relative to the GS as a statistically significant risk factor for miscarriage among SCH patients. Furthermore, the risk of miscarriage showed a progressive increase with higher proportions of SCH within the GS.

## Data Availability

The raw data supporting the conclusions of this article will be made available by the authors, without undue reservation.
