# Supplementary material for: Association between the proportion of subchorionic hematoma within the gestational sac and miscarriage rate in IVF/ICSI patients
Source: Front Med (Lausanne). 2026 Jan 12;12:1630213. doi: 10.3389/fmed.2025.1630213 (PMC12833371; doi:10.3389/fmed.2025.1630213)
Supplement: Supplementary file 1 [file Table_1.DOCX]

Supplementary Table 1 Baseline Characteristics of patients before propensity score matching

| Characteristics | SCH | No SCH | *P* |
| --- | --- | --- | --- |
| n | 358 | 347 |  |
| Age | 31.8 ±4.1 | 32.5 ±4.3 | 0.02 |
| Infertility type |  |  | 0.13 |
| Primary infertility | 194(54.2) | 168(48.4) |  |
| Secondary infertility | 164(45.8) | 179(51.6) |  |
| Infertility duration | 4.2 ±2.7 | 4.2 ±3.0 | 0.94 |
| Number of abortions | 0.5±0.9 | 0.6±0.9 | 0.14 |
| Etiology |  |  | 0.77 |
| Unexplained infertility | 8(2.2) | 9(2.6) |  |
| PCOS | 13(3.6) | 12(3.5) |  |
| Recurrent IUI failure | 8(2.2) | 7(2.0) |  |
| Low ovarian reserve | 11(3.1) | 22(6.3) |  |
| Male factor | 41(11.5) | 33(9.5) |  |
| Ovulatory dysfunction | 4(1.1) | 4(1.2) |  |
| Tubal factor | 108(30.2) | 99(28.5) |  |
| Sexual intercourse disorders | 0 | 1(0.3) |  |
| Endometriosis | 9(2.5) | 7(2.0) |  |
| Genetic factor | 1(0.3) | 2(0.6) |  |
| Multiple factors | 155(43.3) | 151(43.5) |  |
| AMH | 4.2±4.3 | 4.2±3.8 | 0.97 |
| Basal FSH | 6.0±2.6 | 6.3±3.1 | 0.16 |
| Type of cycle |  |  | 0.12 |
| Fresh cycle | 99(27.7) | 73(27.7) |  |
| Frozen cycle | 257(71.8) | 274(79.0) |  |
| Mixed cycle | 2(0.7) | 0 |  |
| Type of embryo transferred |  |  | 0.03 |
| D3 | 140(39.1) | 108(31.1) |  |
| D5 | 218(60.9) | 239(68.9) |  |
| Number of embryos transferred | 1.7±0.5 | 1.6±0.5 | 0.21 |
| Grade of embryo transferred |  |  | 0.87 |
| A | 303(84.6) | 296(85.3) |  |
| B | 40(11.2) | 35(10.1) |  |
| C | 15(4.2) | 16(4.6) |  |

“mixed cycle" refers to a treatment cycle in which, following egg retrieval, both thawed frozen embryos and fresh embryos are transferred simultaneously.
